# Oral Administration Evaluation of the Hydro-Ethanolic Extract of Ginger (Rhizome of *Zingiber officinale*) against Postoperative-Induced Peritoneal Adhesion: Investigating the Role of Anti-Inflammatory and Antioxidative Effects

**DOI:** 10.1155/2023/4086631

**Published:** 2023-02-21

**Authors:** Roghayeh Yahyazadeh, Vafa Baradaran Rahimi, Seyed Ahmad Mohajeri, Milad Iranshahy, Ahmad Yahyazadeh, Maede Hasanpour, Mehrdad Iranshahi, Vahid Reza Askari

**Affiliations:** ^1^Department of Pharmacodynamics and Toxicology, School of Pharmacy, Mashhad University of Medical Sciences, Mashhad, Iran; ^2^Applied Biomedical Research Center, Mashhad University of Medical Sciences, Mashhad, Iran; ^3^Department of Cardiovascular Diseases, Faculty of Medicine, Mashhad University of Medical Sciences, Mashhad, Iran; ^4^Department of Pharmacognosy, School of Pharmacy, Mashhad University of Medical Sciences, Mashhad, Iran; ^5^Department of Histology and Embryology, Faculty of Medicine, Karabuk University, Karabuk, Turkey; ^6^Biotechnology Research Center, Pharmaceutical Technology Institute, Mashhad University of Medical Sciences, Mashhad, Iran; ^7^International UNESCO Center for Health-Related Basic Sciences and Human Nutrition, Mashhad University of Medical Sciences, Mashhad, Iran; ^8^Pharmacological Research Center of Medicinal Plants, Mashhad University of Medical Sciences, Mashhad, Iran

## Abstract

Peritoneal adhesions (PAs) occur and develop after abdominal surgery. Abdominal adhesions are common and often develop after abdominal surgery. Currently, there are no effective targeted pharmacotherapies for treating adhesive disease. In this regard, ginger is wildly used in traditional medicine because of its anti-inflammatory and antioxidant effects and has been investigated for peritoneal adhesion treatment. This study analyzed ginger ethanolic extraction via HPLC to have a 6-gingerol concentration. Four groups induced peritoneal adhesion to evaluate ginger's effects on peritoneal adhesion. Then, ginger extract (50, 150, and 450 mg/kg) was administered by gavage in various groups of male Wistar rats (220 ± 20 g, 6–8 weeks). After scarifying the animals for biological assessment, macroscopic and microscopic parameters were determined via scoring systems and immunoassays in the peritoneal lavage fluid. Next, the adhesion scores and interleukin IL-6, IL-10, tumor necrosis factor-(TNF-) *α*, transforming growth factor-(TGF-) *β*1, vascular endothelial growth factor (VEGF), and malondialdehyde (MDA) were elevated in the control group. The results showed that ginger extract (450 mg/kg) notably decreased inflammatory (IL-6 and TNF-*α*), fibrosis (TGF-*β*1), anti-inflammatory cytokine (IL-10), angiogenesis (VEGF), and oxidative (MDA) factors, while increased antioxidant factor glutathione (GSH), compared to the control group. These findings suggest that a hydro-alcoholic extract of ginger is a potentially novel therapeutic strategy for inhibiting adhesion formation. Also, it might be considered a beneficial anti-inflammatory or antifibrosis herbal medicine in clinical trials. However, further clinical studies are required to approve the effectiveness of ginger.

## 1. Introduction

Peritoneal adhesions (PAs), with an estimated incidence rate of around 66% in surgeries worldwide [1], are caused by trauma, abdominal surgery, intraperitoneal (IP) infections [2], and various factors related to the types of operation, organs undergoing surgery, and suture types [3]. As a substantial reason, peritonitis and open procedure (laparotomy) can trigger PA progression. Also, various surgical and operation approaches are directly related to PA formation [4, 5]. Statistically, in the UK, a study on 72,270 patients who underwent different types of surgery showed that 17.6% of them were estimated for disorders related to adhesion, 13.1% for surgical operations possibly caused by adhesions, and 3.5% for problems directly accompanied by adhesions [6]. In addition, PA can be a reason for female infertility [7], intestinal obstruction, and further chronic abdominal complications [3, 8].

Recently, natural products and herbal medicine have been suggested to prevent and treat PA after the operation in animal models [9–15]. The beneficial effects of such products and medicine are attributed to their antioxidant and anti-inflammatory characteristics [9, 16]. Pathophysiology of PA is correlated with increased inflammatory cytokines (IL-6, TNF-*α*, and PGE2), fibrin markers (TGF-*β*1, IL-4, and plasminogen activator inhibitor (PAI)), angiogenesis markers, and matrix metalloproteinase (MMPs). These agents are secreted from mesothelial cells, fibroblasts, and immune cells in the adhesion site. Overall, PA expansion is stimulated through mentioned mediators by interfering with the fibrinolytic pathways, elevating the proliferation of endothelial cells, promoting angiogenesis, and changing the fibrinogen matrix [10, 14].

Monocytes, leukocytes, and endothelial cells trigger the initiation of inflammation. As a result, inflammation mediators are associated with adhesion molecules, such as integrin, selectins, and immunoglobulins (Ig), including a supergene family of proteins such as intercellular adhesion molecules (ICAMs) and vascular cell adhesion molecules (VCAMs) [12, 17–21]. In this regard, some preclinical studies have shown that inhibiting LFA-1/ICAM-1-mediated cell adhesion, as novel therapeutic agents, could be considered to treat inflammatory diseases such as autoimmune disease, collagen-induced arthritis, and models of transplantation [22, 23]. Furthermore, repairing the peritoneal injury occurs after PA formation. Hence, this knowledge helps determine which compounds can be used against PA regarding their intrinsic properties (e.g., immunogenicity, biodegradability, interference in the peritoneum repair procedure, and lifetime). These properties might eventually induce anti-PA efficacy [9–15, 24]. The ideal anti-PA compound increases or even removes adhesions without affecting wound healing. PA formation is directly dependent on coagulation, inflammation, and fibrinolysis. Therefore, to reach the aim of prevention and treatment of adhesions, we should omit profibrotic signals and decrease the extreme accumulation of fibrosis [25].

Ginger, the rhizome of *Zingiber officinale* Roscoe, has been consumed in food as a supplement, spice, and flavoring agent. The medicinal use history of ginger dates back to 2500 years ago in China, Iran, and India for some disorders, such as nausea, diarrhea, vomiting, dyspepsia, rheumatism, colds, and flu [26, 27]. Also, it is commonly used for its beneficial characteristics such as aroma, nutrients, pungency, and pharmacological activities. Moreover, its rhizome has beneficial effects on diabetes [28] and metabolic syndrome [27], cholesterol levels [29], lipid metabolism [30], and inflammation (as a replacive target to nonsteroidal anti-inflammatory drugs for osteoarthritis disease conditions) [31, 32]. Ginger has many proven biological (e.g., apoptosis, cell cycle/DNA damage, chromatin/epigenetic regulation, cytoskeletal regulation and adhesion, immunology and inflammation, and neuroscience) and pharmacological properties. The biological and pharmacological properties of ginger, as functional food, dietary supplement, and natural immunomodulatory, are owing to the presence of monoterpenes (cineole, citral, limonene, and *α*/*β*-pinenes), sesquiterpenes (*β*-elemene, farnesene, and zerumbone), and phenolics (gingerols, [6]-Shogaol, [6]-paradol, and zingerone). These properties are induced through specific signaling pathways such as autophagy, mitogen-activated protein kinase, and cellular metabolism [27, 33]. In addition, research has shown that ginger can induce estrogen activity (e.g., the chemoprevention of cancers) and the improvement of menopausal syndromes, osteoporosis, endometriosis, prostatic hyperplasia, and polycystic ovary syndrome [33, 34]. *In vitro* studies have shown that the anti-inflammatory effects of ginger are induced by the inhibition of arachidonic acid metabolism in both lipoxygenase (LOX) and cyclooxygenase (COX) pathways [35], which may show fewer side effects than NSAID [36]. Another study has shown that ginger inhibits the production of the genes participating in the inflammatory reactions (e.g., chemokines, genes encoding cytokines, and the inducible enzyme nitric oxide synthase (iNOS) and COX-II) [27, 37].

The preclinical and clinical surveys have used several natural and synthetic bio-components for PA treatment. However, there still is not enough knowledge to express a “gold standard” for curative effects on postoperative PA. Therefore, surgeons and researchers still investigate impressive new methods [38, 39], although none is extensively applied clinically due to a missing long-term efficacy. Therefore, in the present study, we investigate the protective effects of ginger against surgical-induced peritoneal adhesion in a rat model.

## 2. Materials and Methods

### 2.1. Chemicals and Reagents

Ethanol was purchased from Sigma–Aldrich Chemical Co. (St. Louis, MO, USA). Normal saline (0.9% w/v) was prepared from the Samen® pharmacy factory, Iran. Xylazine and ketamine were prepared from Alfasen, Woerden, Holland. Also, enzyme-linked immunosorbent assay (ELISA) kits, including VEGF, IL-1*β*, IL-6, TGF-*β*, IL-10, and TNF-*α*, were obtained from IBL International® Company, Switzerland. The levels of malondialdehyde (MDA), as an oxidative marker, and glutathione (GSH), as an antioxidative marker, were measured using commercially available biochemistry kits ZellBio®, Germany. Dichloromethane (DCM) was purchased from Caledon, Canada, for HPLC grade. In addition, deionized water and ethanol (96%) were obtained from Abtin Co. (Iran), Kian Kaveh Azma Co. (Iran), and Taghtir Khorasan Co. (Iran), respectively. Furthermore, 6-gingerol was purchased from Golexir Pars ® Co., Iran, as an internal standard. Further chemicals and reagents were from Sigma–Aldrich Chemical Co. (St. Louis, MO, USA).

### 2.2. Plant Material and Extract Preparation

The ginger rhizome was purchased from a local herbal market in Mashhad, Khorasan Razavi province, Iran. The rhizome was authenticated and confirmed by Mr. Joharchi, a faculty member of the medicinal plants and Herbarium Research Institute of the Ferdowsi University of Mashhad. Here confirmed the rhizome as for *Zingiber officinale* Roscoe (herbarium No. E1260-FUMH). After washing with tap water and peeling neatly, the rhizomes were cut with a knife into small pieces. Then, it was soaked finely in ethanol (70 v/v%) for 72 hours. Afterward, the mixture was filtered and sieved. The fresh ginger extraction was evaporated in the incubator at 37°C, yielding a solid powder. This powder is stored in the freezer at −20°C until used. Finally, the extract was dissolved in normal saline containing 5 v/v% Tween 80 [11, 15].

### 2.3. Extract Standardisation Using High-Performance Liquid Chromatography

The high-performance liquid chromatography (HPLC) analysis was performed using a C18 column (5 *μ*m particle size, 250 × 4.6 mm) from Capital (Broxburn, UK) with a 20 *μ*l injection volume. The HPLC analysis system was equipped with a Knauer HPLC system (Berlin, Germany), UV detector K‐2600, HPLC pump (K‐1001), and a Knauer K‐500 degasser. The chromatograms were registered at 280 nm wavelength. The mobile phase included distilled acidified water with trifluoroacetic acid (TFA, 0.05%) and methanol at a 1 ml/min flow rate at the controlled room temperature (22–25°C). We prepared a standard stock solution of 6-gingerol (500 *μ*g/ml) in methanol. It was then diluted with the same solvent to make 100 and 50 *μ*g/ml concentrations for quantitative analysis, and the standard curves were plotted. A linear gradient was used as follows: at 0 min (80% solvent A and 20% solvent B); at 7 min (100% solvent); 7–11 min (100% solvent B); at 13 min (80% solvent A and 20% solvent B); and 13–15 min (returning to initial conditions).

### 2.4. Animals and Treatments

#### 2.4.1. Ethical Statements and Animal Rearing

Ethical and basic principles of use and animal care were complied with according to the institutional guidelines from the Medical University of Mashhad University, Iran (Approval date: 20-10-2020, ethical approval code: IR.MUMS.MEDICAL.REC.1399.486, approval ID: 990614, approval date: 25-11-2020).

In this study, 40 healthy mature male Wistar albino rats with weights of 250 ± 30 g and ages of 6–8 weeks were purchased from the animal laboratory of the Faculty of Medicine, Mashhad University of Medical Sciences, and Mashhad, Iran. For adaptation, they were acclimatized to the laboratory conditions and had *ad libitum* access to tap water and diet for one week before starting the experiments. In addition, more appropriate hygiene was provided with continuous cleaning and removal of feces and spilled feeds from cages daily. The experimental rats were all alive until being sacrificed with the asphyxia method using a CO_2_ chamber. All animals received human care in compliance with institutional guidelines.

#### 2.4.2. Experimental Design and Interventions

The rats were randomly and equally divided into five groups, each with 8 rats, as follows:

- Group 1: sham group underwent surgical procedures but was not induced by PA.
- Group 2: control group underwent surgical procedures, and PA was induced.
- Groups 3 to 5: ginger groups underwent surgical procedures, and PA was induced. Next, the groups received the oral administration of the ginger extract (50, 150, and 450 mg/kg), respectively, for seven constitutive days.

According to our previously published articles [10, 11, 14, 15], the experimental procedure was performed as follows: after anesthetization with 100 mg/kg of ketamine (intraperitoneally; IP) and 10 mg/kg of xylazine (IP) [11, 13–15], the rats' abdomen was shaved. In this process, alcohol and iodine solution was used for skin disinfection. Next, a 3 cm gap was carefully cleaved in the abdominal midline to achieve the abdominal cavity for inducing the adhesion aim. Then, for peritoneal abrasion, one side of the middle abdominal hole was softly abraded via soft sterilized sandpaper until the cecum displayed a fine petechia and turbid appearance. Afterward, 4–0 polyglactin sutures were performed to close the abdomen wall after inducing the adhesion. After ending the surgery to inhibit the possible wound infection, a single dose of antibiotic cefazolin (300 mg/kg intramuscularly; IM, [11, 13, 14]) was immediately given to all rats. Finally, the rats were reserved in their cages for seven days to recover.

#### 2.4.3. Assessment of Adhesion Grade

The laparotomy was performed on day 7 after the surgery. Two scoring systems, i.e., Nair et al. [11, 14, 40] and adhesion scoring scheme [11, 14, 41], were then used to determine PA scores (Tables 1 and 2, respectively), with two independent researchers blinded on the procedures and the grouping.

#### 2.4.4. Preparation of Peritoneal Lavage Fluid

After the rats' laparotomy, the peritoneal lavage fluid was prepared using 2.5 mL sterilized phosphate-buffered saline (PBS). In detail, the whole area of the peritoneum was washed twice. Then, the collected fluid was centrifuged at 3000 RPM for 5 min at 4°C. Finally, the supernatant was separated for further investigation.

#### 2.4.5. Assessment of Immunological and Biochemical Parameters

In this study, inflammatory cytokines (TNF-*α*, IL-6, and IL-1*β*), oxidative biomarkers (NO, MDA, and GSH), an angiogenesis marker (VEGF), and a fibrosis biomarker (TGF-*β*) were evaluated from peritoneal lavage of the sample via ELISA kits according to the manufacturer's instructions [10, 20].

### 2.5. Statistical Analysis

Graph Pad Prism (version 8.01) software was used for data analysis according to the nature of parametric or nonparametric analysis and expressed as means ± SD or median ± IQR. Brown-Forsythe one-way analysis of variance (ANOVA) was performed via Dunnett's T3 multiple comparisons post hoc test for parametric data (Table 3). Also, for the nonparametric data (adhesion scores), a Kruskal–Wallis test was utilised via Dunn's multiple comparison post hoc test. Data of wound size were then analyzed using repeated-measurement two-way ANOVA with Dunnett multiple comparisons post hoc tests. *P* values *P* ≤ 0.01 were considered statistically significant [20, 21].

## 3. Results

### 3.1. HPLC Analysis of the Ginger Extract

**Figure 1:**
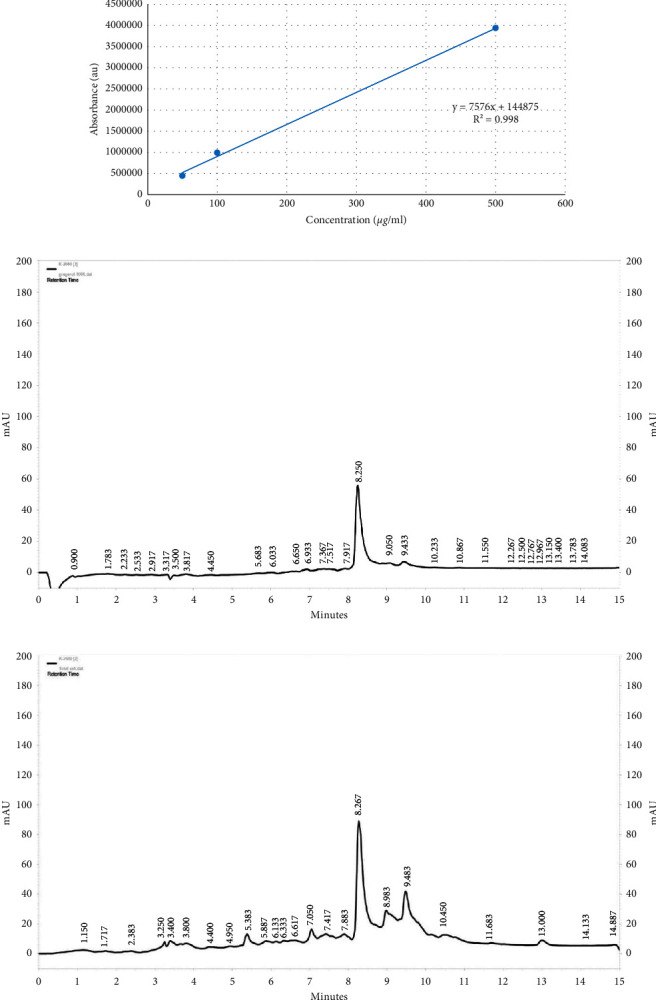
(a) Standard curve of 6-gingerol in 500, 100 and 50 *μ*g/ml concentrations; (b) chromatogram of 6-gingerol with a 100 *μ*g/ml concentration at 280 nm; (c) chromatogram of *Zingiber officinale* extract with a concentration of 5 mg/ml at 280 nm.

HPLC is the most standard application method for the quality control and analysis of ginger and ginger-related marketed products [42, 40]. This method is performed for quantitative and qualitative analysis of pungent ginger principles. First, the peak areas (*y*) against the standard solution (*x*) concentration are plotted, and the calibration curve equation is calculated as follows: *y*=7576*x*+144875, with *R*^2^ = 0.998 (Figure 1(a)). Then, the extract solution was prepared in methanol with a concentration of 5 mg/ml and injected into the HPLC-diode array detection-evaporative light scattering system for analysis (Figures 1(b) and 1(c)). All standard samples and extract samples were analyzed in triplicate. The concentrations of the 6-gingerols were calculated using the plotted calibration curve (Figure 1(a)). Also, the content of 6-gingerol was calculated at 39.05 mg in 1 g of the dried extract (Figures 1(b) and 1(c)).

### 3.2. Evaluating Ginger Extract Effects on the Body and Spleen Weights and Wound Healing

**Figure 2:**
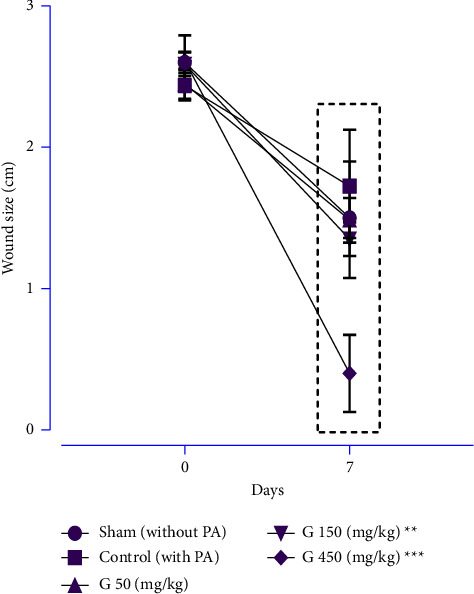
The effects of different doses of ginger extract on wound healing. All data were presented as means ± SD (*n* = 8 for each group). Data were analysed using repeated-measurement two-way ANOVA with Dunnett multiple comparison post hoc tests. ^*∗*^internally compared the wound size between days zero and seven of each group; ^*∗∗*^: *P* < 0.01 and ^*∗∗∗*^: *P* < 0.001.

This experiment was conducted to evaluate the effects of ginger extraction on the rat's body and spleen weights (Table 4), spleen size (Table 4), and wound healing (Figure 2). Ginger extract with 50, 150, and 450 mg/kg doses had no significant effect on body and spleen weights compared with the control and sham groups at the end of the study period. However, the wound size of groups treated with 150, 450 mg/kg was significantly smaller than at the beginning of the study (Figure 2; *P* < 0.001 for all cases). However, the ginger extract had a remarkable effect on wound healing compared with the control group at the end of during (Figure 2; *P* < 0.01 and *P* < 0.001, respectively).

### 3.3. Evaluating Ginger Extract Effects on Peritoneal Adhesion (PA) Scoring

**Figure 3:**
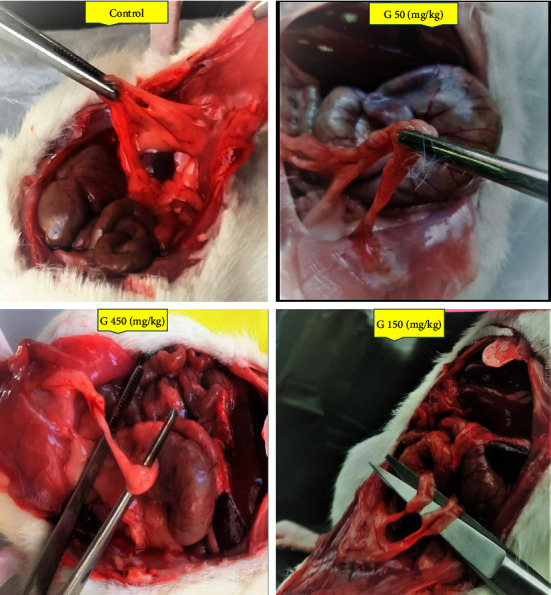
Macroscopic illustration of peritoneal adhesions bands after seven days, in control, and ginger extract at doses of 50, 150, and 450 mg/kg groups.

**Figure 4:**
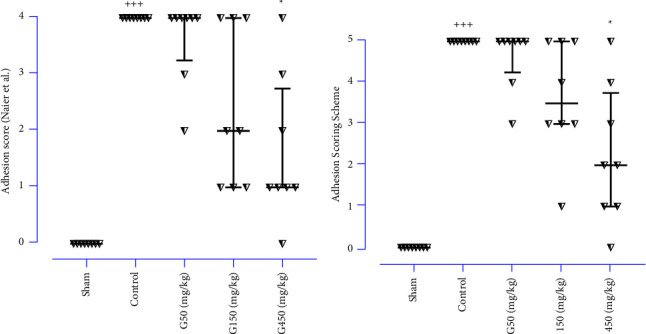
The effects of various doses of ginger extract on adhesion scores were evaluated by (a) Naier et al. and (b) adhesion scoring scheme. Scoring systems; data were presented as median ± interquartile range, IQR (*n* = 8 for each group). Data were analysed using the kruskal-wallis test with Dunn's multiple comparison post hoc test. ^*∗*^compared the treated group with the control group; ^*∗*^: *P* < 0.05. ^+^compared the control group with the sham group; ^+++^: *P* < 0.001.

At the end of the experiment, the incisions of all rats were primarily ameliorated without infection or other complications. Peritoneal adhesions are represented in Figure 3. The results demonstrated that the PA scores were significantly elevated in the control group compared to the sham group (Figure 4; *P* < 0.001 for both scoring systems: Naier et al. and adhesion scheme scoring). Meanwhile, 450 mg/kg of ginger markedly reduced the PA scores compared to the control group (Figure 4; *P* < 0.05 for both scoring systems).

### 3.4. Evaluating Ginger Extract Effects on Inflammatory and Anti-inflammatory Biomarkers

**Figure 5:**
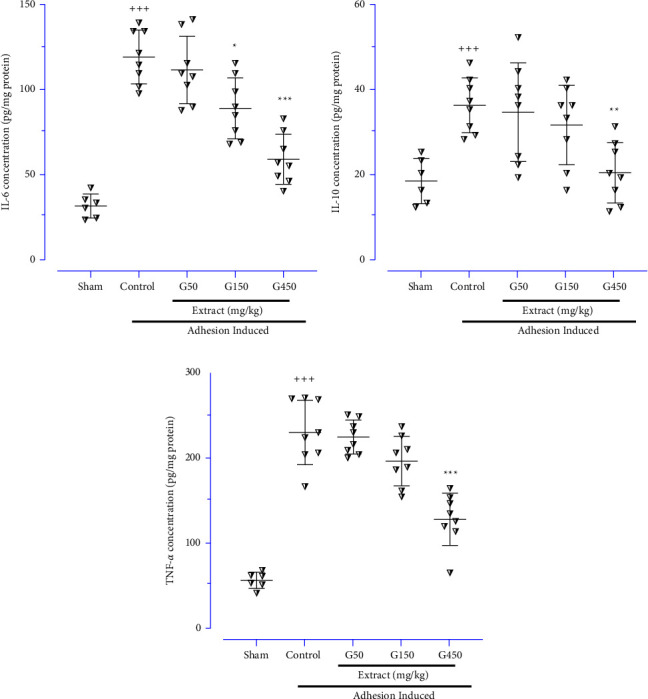
The effects of different doses of ginger extract on the peritoneal lavage levels of (a) IL-6, (b) IL-10 and (c) TNF-*α*; data were obtainable as means ± SD (*n* = 6–8 for each group). Brown-Forsythe one-way analysis of variance (ANOVA) was utilised via Dunnett's-T3 multiple comparisons post hoc test for parametric data. ^*∗*^Compared the treated group with the control group; ^*∗*^: *P* < 0.05, ^*∗∗*^: *P* < 0.01 and ^*∗∗∗*^: *P* < 0.001. ^+^Compared the control group with the sham group; ^+++^: *P* < 0.001.

Our results showed that the levels of inflammatory (IL-6, Figure 5(a), and TNF-*α*, Figure 5(c)) and anti-inflammatory (IL-10, Figure 5(b)) biomarkers were meaningfully elevated in the control group compared to the normal group (*P* < 0.001 for all cases). The ginger extract at both higher doses (150 and 450 mg/kg) significantly decreased the level of IL-6 (Figure 5(a); *P* < 0.05 and *P* < 0.001, respectively). However, 450 mg/kg of ginger extract notably reduced the levels of IL-10 (Figure 5(b); *P* < 0.01) and TNF-*α* (Figure 5(c); *P* < 0.001) compared with the control group.

### 3.5. Evaluating Ginger Extract on Fibrosis and Angiogenesis Parameters

**Figure 6:**
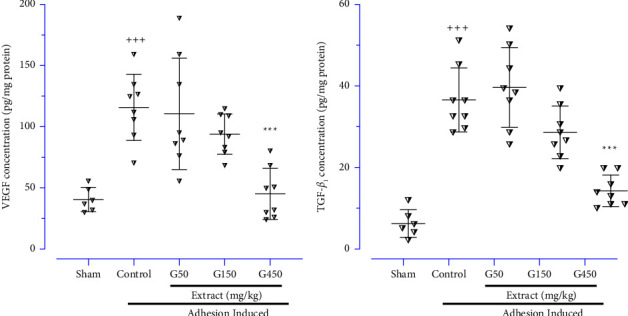
The effects of different doses of ginger extract on the peritoneal lavage levels of (a) VEGF and (b) TGF-*β*; data were obtainable as means ± SD (*n* = 6–8 for each group). Brown-Forsythe one-way analysis of variance (ANOVA) was utilised via Dunnett's-T3 multiple comparisons post hoc test for parametric data. ^*∗*^Compared the treated group with the control group; ^*∗∗∗*^: *P* < 0.001. ^+^ Compared the control group with the sham group; ^+++^: *P* < 0.001.

Following the PA, the angiogenesis and fibrosis levels were notably increased by determining VEGF (Figure 6(a)) and TGF-*β*1 (Figure 6(b)), respectively, in the control group compared to the sham group (*P* < 0.001 for both cases). Nevertheless, the ginger extract at the dose of 450 mg/kg significantly diminished the levels of VEGF (Figure 6(a)), and TGF-*β*1 (Figure 6(b)) compared to the control group (*P* < 0.001 for both cases).

### 3.6. Evaluating Ginger Extract on Oxidant and Antioxidant Parameters

**Figure 7:**
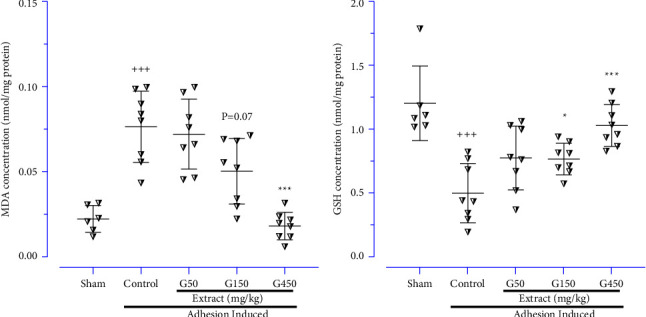
The effects of different doses of ginger extract on the peritoneal lavage levels of (a) MDA and (b) GSH; data were presented as means ± SD (*n* = 6–8 for each group). Brown-Forsythe one-way analysis of variance (ANOVA) was utilised via Dunnett's-T3 multiple comparisons post hoc test for parametric data. ^*∗*^Compared the treated group with the control group; ^*∗*^: *P* < 0.05 and ^*∗∗∗*^: *P* < 0.001. ^+^Compared the control group with the sham group; ^+++^: *P* < 0.001.

As an oxidative marker, the MDA level was significantly increased in the control group compared to the normal group (Figure 7(a); *P* < 0.001). In contrast, the results indicated that the GSH level, as an antioxidative marker, was strongly down-regulated in the control group compared to the normal group (Figure 7(b); *P* < 0.001). Nevertheless, administration of the ginger extract (450 mg/kg/day) remarkably decreased the MDA level (Figure 7(a)) and increased the GSH level (Figure 7(b)) compared to the control group (*P* < 0.001 for both cases). In addition, the ginger extract (150 mg/kg/day) could significantly enhance the GSH level compared to the control group (Figure 7(b); *P* < 0.05).

## 4. Discussion

The main finding of our study was that ginger extract could lower the PA following surgeries. To the best of our knowledge, this is the first report describing the significance of the ginger effect on PA from both clinical medicine and basic science viewpoints. Our results suggested that ginger extract may be considered a herbal medicine that can improve the PA in patients by suppressing the inflammatory cytokines such as IL-6 and TNF-*α*, and TGF-*β*1 and VEGF as fibrotic and angiogenesis factors. In this study, the ginger extract reduced the upregulated-IL-10 in the control group. Also, it has been shown that ginger extract had a curative effect on macroscopic parameters (wound healing). However, it was not effective on the body and spleen weight change, despite increasing spleen weight in the broiler chickens [43]. In addition, it could increase the weight change in Japanese quail [44]. Regarding these discrepancies, further research is needed for the conclusion.

Local injuries (e.g., peritoneal surgery) lead to a growth in the number and infiltration of leucocytes in the bloodstream to the injured area. As a result, inflammatory cytokines such as TNF-*α*, IL-1*β*, IL-6, and interferon (IFN)-*γ* are overproduced and majorly appear in both bloodstream and the injured area [11, 45, 46]. Moreover, infections and tissue injuries cause temporary secretion of IL-6, thereby simulating acute phase responses, hematopoiesis, and immune reactions. However, IL-6 expression is tightly regulated by transcriptional and posttranscriptional mechanisms, and uncontrolled synthesis of IL-6 shows a pathological effect on chronic inflammation and immunity [47]. In line with this result, ginger extract at 50 mg/kg remarkably attenuated the serum levels of TNF-*α*, IL-6, and IL-17 in the ginger group than in the type 2 collagen and Freund's adjuvant-induced arthritis group [48]. Also, Adegbola et al. demonstrated that the ginger extract at a dose of 500 mg/kg alone or in combination with *Allium sativum* 1000 mg/kg significantly declines the inflammation following the high-fat diet-induced obesity in rats by decreasing the IL-6 and TNF-*α* levels and lipid profile [49]. Another study showed that oral administration of *Zingiber officinalis* extract at doses of 100 and 1000 mg/kg strongly diminishes LPS-Induced Inflammation by downregulating the release of proinflammatory cytokines such as IFN-*γ* and IL-6 [50]. These studies could support our findings regarding the anti-inflammatory effects of ginger extract.

Furthermore, IL-6 facilitates the differentiation of naïve CD4^+^ T cells. Therefore, it plays an essential function in relating innate to acquired immune responses. In addition, IL-6, incorporated with TGF-*β*, is critical for Th17 differentiation from naïve CD4^+^ T cells [51]. Research has shown that the activation of IL-6 signaling entirely relates to the presence of sIL-6R in malignant mesothelioma cells [52]. As a result, the signal transduction cascade is initiated by IL-6. TGF-*β*1, secreted from most immune cells, is essential in regulating the immune system [53]. Also, muscle injury causes the expression of TGF-*β*1, which triggers chronic inflammation, fibrosis, and extracellular-matrix accumulation [54]. TNF-*α*, as a proinflammatory cytokine, activates intracellular signaling pathways (e.g., MAPKs, transcription factors, or apoptosis) that contribute to vasodilatation and leukocyte adhesion to epithelium through the effect on adhesion molecules expression. IL-10, as an anti-inflammatory biomarker, is secreted by various cells (T cells, B cells, and monocytes/macrophages) triggered by innate immune cells. Besides, it lowers the concentration levels of IL-6, IL-8, and TNF-*α* [55].

Therefore, neutralizing TGF-*β*1 and TNF-*α* expression in injured tissue could inhibit the formation of inflammation and fibrosis [54, 56]. Consistent with our findings, Abdi et al. showed that the ginger extract at doses of 100, 200, and 400 mg/kg provides sufficient cardioprotection in the diabetic group by reducing inflammatory mediators and decreasing the fibrotic markers gene expression, including angiotensin II type 1 receptor and TGF-*β*1 and TGF-*β*3 [57]. Furthermore, the extract induces its effect by regulating the SMAD/TGF-*β* signaling pathway [57].

The STAT3 signaling pathway induces the VEGF by stimulating IL-6 in C22A cervical carcinoma cell lines [58]. The increased VEGF levels caused by IL-6/sIL-6R are related to the STAT3 pathway. Hence, IL-6 can trigger VEGF and vascularisation in chronic inflammatory diseases [59]. Moreover, a recent study demonstrated that ginger constituent 6-Shogaol inhibits the development of vascular endothelial growth factor (VEGF)-induced endothelial blossoms from human umbilical vein endothelial cells (HUVECs) spheroids and from murine aortic rings. Importantly, for the first time, this research shows that 6-Shogaol disrupts aortic angiogenic sprouts in a murine model of vascular disruption [60]. Another recent study investigated the potential protective effects of ginger extract on rats with type 2 diabetic retinopathy. The results showed that diabetic rats had altered ocular expression of e/iNOS, G6PDH, VEGF, NF-kB, and apoptosis-related genes. In contrast, ginger extract has been shown to reduce oxidative damage, inflammation, iNOS, VEGF, and apoptosis and improve eNOS and G6PDH in diabetic retinopathy. These results also are consistent with our findings [61].

This study showed a considerable difference in MDA and GSH in ginger-treated rats. Our results are similar to the survey reported by some researchers [13, 62, 63]. The effect of ginger on lipid peroxidation is applied via Fe^2+^ chelating properties, antioxidant activity, and OH scavenging ability. The ginger administration has been displayed to strongly downregulate thiobarbituric acid-reactive substances (TBARS) levels, which expresses as an indicator of lipid peroxidation and oxidative stress damage. Accordingly, reducing TBARS levels upregulates the GPX function and impact, thereby inactivating the lipid peroxidation reactions [64]. ROS is generated in the postoperative site or inflammatory diseases. Consequently, an antioxidant enzyme (e.g., catalase, superoxide dismutase (SOD), GSH, and glutathione peroxidase (GPx)) is needed to remove ROS [65, 66]. The GSH, as a nonprotein thiol, is a vital need for protecting cells from the cytotoxic effect of ROS. Also, the harmful effects of As were suppressed via GSH by elevating the converting the methylation form of As to low-toxic metabolites [67]. Many *in vitro*, *in vivo*, and clinical studies have proven the safety of ginger and shown that it has no adverse/toxic effects [68–71]. In a clinical study, patients associated with chemotherapy-induced nausea and vomiting (CINV) who used the ginger extract (40 mg/day) showed no further gastrointestinal disorders such as epigastric and abdominal pain, dyspepsia, and hiccups than the placebo group [68]. Moreover, it has been suggested that using the standardized ginger extract up to 2 g/day leads to no notable adverse effects on human health. Therefore, it is classified as first ranked on the toxicity scale of the National Cancer Institute [72]. In addition, clinical studies have demonstrated that ginger and its extract (up to 1.5 g/d) can be consumed to attenuate nausea and vomiting during early pregnancy [73].

In summary, this study demonstrated that oral ginger intake induced beneficial effects on postoperational adhesion without any adverse effects by improving the antioxidative factors and decreasing the fibrosis, inflammatory cytokines, oxidative factors, and angiogenesis biomarkers. Hence, ginger extraction can be used to treat postoperative PA as a potential herbal medicine. However, further clinical studies are required to approve the effectiveness of ginger.

## Figures and Tables

**Table 1:** According to Nair et al. [11, 14, 40], the adhesion score is categorised.

Grade	Description of adhesive bands
0	The complete absence of adhesions
1	A single band of adhesion between viscera or from viscera to the abdominal wall
2	Two bands, either between the viscera or from the viscera to the abdominal wall
3	More than two bands, between viscera or viscera to the abdominal wall or whole intestines form a mass without being adherent to the abdominal wall
4	Viscera directly adherent to the abdominal wall, irrespective of the number and extent of adhesive bands

**Table 2:** Adhesion scoring scheme has a scoring system for peritoneal adhesion [11, 14, 41].

Grade	Description of adhesive bands
0	Absence of adhesions
1	A thin layer adhesion
2	More than a thin layer adhesion
3	Thick adhesive tissue attached to the surgical site
4	Thick adhesive tissue attached to different areas of the abdomen
5	Thick adhesive tissue containing blood vessels or too much adhesive tissue or organ adhesive tissue

**Table 3:** ANOVA table for all immunological and biochemical parametric data.

	Groups	*F* ^*∗*^ (DFn, DFd)	Means ± SD	*P* value	Type of ANOVA test
IL-6		38.61 (4.000, 28.74)		*P* < 0.001	Brown-Forsythe-Dunnett's-T3
	Sham		31.17 ± 7.13	*P* < 0.0001	
	Control		119.6 ± 15.97	—	
	G 50 mg/kg		112 ± 20.02	Not	
	G 150 mg/kg		89.13 ± 18.11	*P* < 0.05	
	G 450 mg/kg		58.88 ± 14.88	*P* < 0.0001	

IL-10		7.306 (4.000, 26.50)		*P* < 0.001	Brown-fForsythe-Dunnett's-T3
	Sham		18.17 ± 5.345	*P* < 0.001	
	Control		36 ± 6.459	—	
	G 50 mg/kg		34.38 ± 11.64	Not	
	G 150 mg/kg		31.38 ± 9.334	Not	
	G 450 mg/kg		20.13 ± 7.14	*P* < 0.01	

TNF-Α		50.97 (4.000, 25.25)		*P* < 0.001	Brown-Forsythe-Dunnett's-T3
	Sham		54 ± 9.633	*P* < 0.0001	
	Control		229 ± 38.15	—	
	G 50 mg/kg		223.5 ± 20.08	Not	
	G 150 mg/kg		195.1 ± 29.31	Not	
	G 450 mg/kg		126.39 ± 30.91	*P* < 0.001	

TGF-*β*1		32.84 (4.000, 23.74)		*P* < 0.0001	Brown-Forsythe-Dunnett's-T3
	Sham		6.167 ± 3.488	*P* < 0.0001	
	Control		37.13 ± 8.026	—	
	G 50 mg/kg		40.25 ± 9.982	Not	
	G 150 mg/kg		29 ± 6.59	Not	
	G 450 mg/kg		14.38 ± 3.962	*P* < 0.0001	

VEGF		13.29 (4.000, 17.88)		*P* < 0.001	Brown-Forsythe-Dunnett's-T3
	Sham		39.67 ± 10.07	*P* < 0.0001	
	Control		116 ± 27.41	—	
	G 50 mg/kg		110.8 ± 46.20	Not	
	G 150 mg/kg		93.88 ± 16.69	Not	
	G 450 mg/kg		44.38 ± 21.14	*P* < 0.0001	

MDA		20.69 (4.000, 25.07)		*P* < 0.001	Brown-Forsythe-Dunnett's-T3
	Sham		0.0215 ± 0.00796	*P* < 0.0001	
	Control		0.0764 ± 0.21	—	
	G 50 mg/kg		0.0720 ± 0.0208	Not	
	G 150 mg/kg		0.0499 ± 0.0195	*P* < 0.05	
	G 450 mg/kg		0.0172 ± 0.008	*P* < 0.0001	

GSH		10.56 (4.000, 22.51)		*P* < 0.001	Brown-Forsythe-Dunnett's-T3
	Sham		0.4941 ± 0.235	*P* < 0.0001	
	Control		1.203 ± 0.296	—	
	G 50 mg/kg		0.773 ± 0.253	Not	
	G 150 mg/kg		0.765 ± 0.126	Not	
	G 450 mg/kg		1.032 ± 0.166	*P* < 0.0001	

Dfn: the degree of freedom for the numerator of the *F* ratio; DFd: degrees of freedom denominator; IL: Interleukin; TNF-*α*: tumour necrosis factor-alpha; VEGF: vascular endothelial growth factor; MDA: malondialdehyde; GSH: glutathione; TGF-*β*1: transforming growth factor beta 1. *P* value shows compared with control groups.

**Table 4:** The effects of different doses of ginger extract on the body and spleen weights.

	Ginger extract					
	Day	Sham	Control	50 mg/kg	150 mg/kg	450 mg/kg
Body weight (g)	0	260 ± 18.142	251.12 ± 18.09	237.87 ± 12.54	238.12 ± 6.357	253.75 ± 11.53
	8	258.875 ± 20.32	252.125 ± 23.77	237.75 ± 9.80	239.625 ± 8.86	243.75 ± 12.56
Spleen size (cm)	8	3.913 ± 0.2532	4.075 ± 0.2915	3.938 ± 0.3204	4.038 ± 0.1506	3.913 ± 0.2031
Spleen weight (mg)	8	961.8 ± 145.1	864.0 ± 173.1	857.0 ± 195.4	855.4 ± 78.29	938.8 ± 83.74

Data were presented as means ± SD.

## Data Availability

The data used to support the findings of this study are available from the corresponding author upon reasonable request.
